# Changes in Corneal Basal Epithelial Phenotypes in an Altered Basement Membrane

**DOI:** 10.1371/journal.pone.0014537

**Published:** 2011-01-14

**Authors:** I-Jong Wang, Ray Jui-Fang Tsai, Lung-Kun Yeh, Ryan Yao-Nien Tsai, Fung-Rong Hu, Winston W. Y. Kao

**Affiliations:** 1 Department of Ophthalmology, National Taiwan University Hospital, Taipei, Taiwan; 2 Taipei Eye Center, Taipei, Taiwan; 3 Department of Ophthalmology, Taipei Medical University Hospital, Taipei, Taiwan; 4 College of Medicine, Graduate Institute of Clinical Medicine, Taipei Medical University, Taipei, Taiwan; 5 Department of Ophthalmology, College of Medicine, Chang-Gung Memorial Hospital at Linko, Chang-Gung University, Taoyuan, Taiwan; 6 Department of Ophthalmology, University of Cincinnati Medical Center, Cincinnati, Ohio, United States of America; University of Reading, United Kingdom

## Abstract

**Background:**

To examine the corneal epithelial phenotype in an altered basement membrane.

**Methodology/Principal Findings:**

Corneas from 9 patients with symptoms of continuous unstable corneal curvature (CUCC) were harvested by penetrating keratoplasty and subjected to histology examination and immunohistochemical staining with transactivating and N-terminally truncated pP63 transcript (ΔNp63), cytokeratin 3 (Krt3), ATP-binding cassette sub-family G member 2 (ABCG2), connexin 43 (CX43), p38 mitogen-activated protein kinases (p38MAPK), activating protein 2 (**TFAP2**), and extracellular signal-regulated kinase (**Erk1/2**) monoclonal antibodies. Positive immunostaining with ABCG2, p38MAPK, and **TFAP2** monoclonal antibodies was observed in the basal epithelial cells of CUCC patients, and CX43 and ΔNp63 were detected in the full-thickness epithelial cells of CUCC patients.

**Conclusions/Significance:**

Our results indicate that alteration of the corneal basement membrane induces a de-differentiation-like phenotype in corneal basal epithelial cells.

## Introduction

The corneal epithelium, which forms the anterior protective surface, consists of stratified the squamous epithelium and its underlying intact basement membrane (BM). The corneal epithelium undergoes continuous desquamation; it is later replenished both by apical migration of transient amplifying cells (TACs) at the basal layer, which undergo a limited number of divisions, and by the centripetal migration of limbal basal cells that replenish TACs in the central basal layer of the cornea. The localization of corneal stem cells (SCs) in the limbal basal layer was first suggested by Sun *et al*., who showed in 1971 that the limbal palisades of Vogt contain the proliferative cells and maintain the integrity of corneal epithelium.[1] It has been demonstrated that limbal basal cells in this area have characteristics of SC, such as high proliferative capacity *in vitro* and slow-cycling [^3^H]TdR- or BrdU-labeled cells *in vivo* (as determined in animal studies).[2]–[7] As such, limbal stem cell transplantation (LSCT) has been applied in both clinical and animal studies to repair and/or regenerate the corneal epithelium in eyes that have been traumatized as a result of the destruction of limbal SCs.[8]–[10]

Multiple mechanisms have been proposed for the regulation and maintenance of SCs in the limbus of the cornea. The preferred hypothesis is that adult SCs are regulated by their niche, i.e., a special microenvironment for the maintenance of limbal stem cells in an undifferentiated state, which consists of unique limbal stromal cells and the underlying BM.[11]–[13] The surrounding cells provide a sheltering environment that shields SCs from stimuli that may adversely promote differentiation and apoptosis and threaten the SC reservoir.[14] Notably, the limbus is highly pigmented, due to the presence of melanocytes [1], [15] that have been infiltrated by antigen-presenting Langerhan's cells[16] and suppressor T-lymphocytes[17], and is surrounded by a vascular network.[18] Melanocytes may produce and transport melanin pigments into epithelial cells to minimize damage caused by ultraviolet irradiation, similar to an effect described in the SC-containing bulge area of human skin.[18]

The BM of the limbal epithelium differs from that of the central cornea. For example, the percentage of basal cell membrane occupied by hemidesmosomes was found to be significantly less than that of the central cornea.[19] Unlike that of the cornea, the BM of the limbus is undulating, with papillae or pegs of stroma extending upward[19] and fenestrated[20]–[22] by so-called limbal crypts and focal stromal projections; the central cornea lacks such papillae. The anatomic features present in the limbus suggest that limbal SCs might closely interact with cells in the underlying limbal stroma.[21]–[23] The unique BM structures of the limbal area are constructed as a result of the preferential expression of α9 integrin[24] and N-cadherin[25] without connexin 43[26], suggesting that limbal SCs are influenced by the interaction with the unique extracellular components in the niche. Aside from laminin-1 and laminin-5, the limbal BM also contains laminin α2β2 chains, while the corneal BM does not.[27] Moreover, α1, α2, and α5 chains of type IV collagen are present in the limbal BM, while α3 and α5 chains are present in its corneal counterpart.[27], [28] All of these components might contribute to the distribution of SC in this niche, as has been suggested for intestinal crypt villi.[29] Furthermore, like other SC niches[30], [31] the limbal BM might sequester and, hence, modulate concentrations of growth factors and cytokines that are released from limbal cells in the niche for efficient and precise targeting onto limbal SCs. These observations suggest that the corneal epithelial BM may affect the overlying epithelial phenotype. In animal studies, LSCT over the limbal area for treating limbal deficiency has shown that limbal SCs are able to provide new healthy corneal epithelial cells and restore the lost niches of the stromal layer, thus compensating for the regression of vessels and the rearrangement of stromal lamellae due to limbal SC deficiency. Ultimately, limbal SCs contribute to the repair and regeneration of transparent corneas.[9], [32]

Previous studies[33]–[36] have shown that the amniotic membrane is able to provide a niche environment for limbal SC proliferation and differentiation: limited in number of limbal SCs could be expanded *ex vivo* to become numerous stem/progenitor cells that are p63-positive and BrdU-label retentive but negative for connexin 43 (CX43) and cytokeratin 12 (Krt12). Through *ex vivo* expansion, these SC-like cells can be successfully grown on the human cornea and thereby help to maintain its clarity as well as the homeostasis between corneal epithelium proliferation and differentiation for years.[36] These observations lend further support to the importance of the amniotic membrane as a unique niche environment for the maintenance and expansion of limbal SCs *in vitro*.

The BM of a diseased or traumatized cornea is often disrupted, which subsequently leads to changes in epithelial phenotype. For example, an epithelial plug was once found in the epithelial wound created by radial keratotomy (RK) of the excised corneal buttons obtained after penetrating keratoplasty.[37] Examination of keratoconus corneas by confocal microscopy showed that the epithelial cells assumed a different morphology and phenotype in comparison to normal corneas.[38], [39] Histological examinations have also revealed that the Bowman's membrane and BM are fragmented and disrupted in keratoconus corneas.[40] These findings indicate that epithelial cells in the wound may behave differently from epithelial cells overlying a normal BM and are affected by changes in their environment, e.g., the BM.

In this study, we hypothesize that corneal wounding may modulate the differentiation of corneal epithelial cells due to a disrupted BM, resulting in de-differentiation of corneal epithelial cells, i.e., the likely progenitor cells of corneal epithelium. We studied the phenotype of corneal epithelial cells in the corneas of patients with RK, post-LASIK keratoectasia and keratoconus, among whom BMs differ from those observed in normal corneas.

## Materials and Methods

### Patients

Eight corneas from 9 patients (5 men and 4 women aged 15 to 30 years; mean age = 24.67±5.85 years) were enrolled for these studies (Table 1). The pathological studies were performed preoperatively between January 2001 and August 2008 by one surgeon (Ray JF Tsai) on the excised corneal buttons of those patients receiving penetrating keratoplasty or deep anterior lamellar keratoplasty due to decreased vision or irregular astigmatism. There were 5 eyes with keratoconus, 1 eye with corneal ectasia caused by a previous LASIK surgery and 3 eyes with scars and irregular astigmatism following RK. All of these patients experienced corneal damage and symptoms of continuous unstable corneal curvature (CUCC). We obtained informed consent from all patients. The control corneal tissue section slides were from a 65-year-old male without any ocular disease (gifted by the Cincinnati Eye Bank).

**Table 1:** Clinical manifestations of seven patients with continuously unstable curvatures of corneas (CUCCs) for keratoplasty.

Case No.	Age/Gender	Eye	Clinical diagnosis	Surgery
1	32/F	Left	Post-LASIK ectasia	DALK
2	26/M	Left	Keratoconus	PKP
3	26/F	Left	Keratoconus	PKP
4	15/F	Right	Keratoconus	PKP
5	17/M	Right	Keratoconus	PKP
6	23/M	Both	keratoconus	PKP
7	25/F	Right	RK	DALK
8	30/M	Right	RK	PKP
9	28/M	Right	RK	PKP

PKP = penetrating keratoplasty; DALK = deep anterior lamellar keratoplasty.

### Ethics Statement

This study was approved by the Institutional Review Board of the Taipei Eye Center and adhered to the tenets of the Declaration of Helsinki. Informed consent was obtained from all patients.

### Immunohistochemistry

For the immunofluorescent study, corneal buttons were frozen in OCT compound, and cryostat sections were then cut, mounted on slides, and fixed in 4% paraformaldehyde in PBS. Nonspecific staining was blocked (30 minutes) with blocking buffer (10% normal donkey serum, 1% bovine serum albumin [BSA] in PBS). An immunohistochemical marker for connexin 43 (CX43, rabbit polyclonal antibodies against amino acids 346–360 of the connexin 43 peptide, dilution 1∶100, obtained from Santa Cruz Biotechnology, Santa Cruz, CA, U.S.A.) was then applied and reacted overnight at 4°C. After the slides were washed with PBS, the slides were incubated for 30 minutes with Alexa 488-conjugated anti-rat IgG. Double immunohistochemical staining for CX43-ΔNp63 was performed with mouse monoclonal antibody 4A4 (mAb 4A4) against ΔNp63 (dilution 1∶50, purchased from Chemicon, Temecula, CA, USA). The pair of primary antibodies was applied to the samples simultaneously, and the secondary antibodies (donkey Alexa 488 anti-rat IgG and Alexa 594 anti-mouse IgG antibodies) were then applied after the samples were washed with PBS. All samples were mounted in a fluorescent mounting solution (VectA Mount; Vector Laboratories, Burlingame, CA). Images were obtained with a confocal laser scanning microscope and a fluorescence microscope (Leica, Heidelberg, Germany).

The specimens used for immunohistochemistry on paraffin sections were fixed with 4% paraformaldehyde in phosphate-buffered saline (PBS) at room temperature, embedded in paraffin, and sectioned at 5 µm. In all cases, mouse monoclonal antibody against cytokeratin 3 (Krt3, AE5, dilution 1∶100, Dako, Denmark) and mouse mAb 4A4 against ΔNp63 were used for staining.

One of the CUCC corneas, comprising half of a corneal button from an RK patient, was further studied with immunohistochemical markers for ABCG2 (dilution 1∶50, obtained from Santa Cruz Biotechnology), CX43, **phospho-p38 MAPK (Thr180/Tyr182) (28B10, Mouse mAb #9216,** dilution 1∶25, from Cell Signaling Technology, Beverly, MA, USA), **TFAP2 (transcription factor activating enhancer binding protein 2 beta)** (dilution 1∶400, purchased from Chemicon, Temecula, CA, USA), and **phospho-p44/42 MAPK (Erk1/2) (Thr202/Tyr204) (E10, Mouse mAb #9106,** dilution 1∶100, from Cell Signaling Technology, Beverly, MA, USA). For comparison, two normal corneas were obtained by trephination of normal human eyes obtained from the eye bank. After fixation, slides were blocked with 3.0% (vol/vol) H_2_O_2_ in methanol for 30 minutes, washed with PBS for 30 minutes, and incubated in primary antibodies at 4°C. All antibodies required a pretreatment antigen-enhancing step of heating the slides in 95°C 0.1 mol/L citrate buffer at pH 6.0 for 10 minutes before incubation with the primary antibody. After primary antibody incubations, slides were incubated with biotinylated secondary antibodies (Dako) for 60 minutes and streptavidin/HRP complex (Dako) for 60 minutes at room temperature. Slides treated with primary antibody anti-CD68/HRP complex did not require secondary antibodies and were developed directly. To develop color, slides were incubated in 3-amino-9-ethylcarbazole (Sigma Diagnostics, Inc., St. Louis, MO, USA) or 3′3-diaminobenzidine as a chromogen with 1% (vol/vol) H_2_O_2_ for 15 minutes, followed by counterstaining with hematoxylin acid solution (Sigma Diagnostics, Inc.) for 10 minutes and washing with H_2_0 for 3 minutes. Sections were then dehydrated in ethanol and xylene, mounted in a permanent mounting medium, and covered with glass cover-slips. Negative controls were obtained by omitting the primary antibodies.

## Results

**Figure 1:**
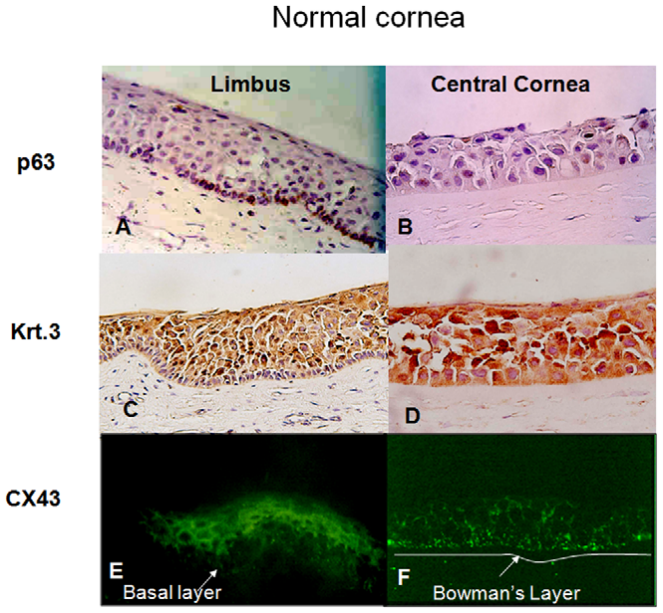
Immunohistochemical study of normal central cornea and limbus with cytokeratin 3 (Krt3), connexin 43 (CX43) and ΔNp63 monoclonal antibodies. In normal limbus (A, C, E), p63 could be detected in the basal epithelial layer (200×, A); Krt3 was detected in the suprabasal epithelial cells (200×, C); CX43 was found in the suprabasal epithelial cells (200×, E). In normal central cornea (B, D, F), no staining of p63 was observed (200×, B); Krt3 could be detected throughout the epithelial cells (200×, D); CX43 was also expressed throughout the epithelial cells (200×, F).

**Figure 2:**
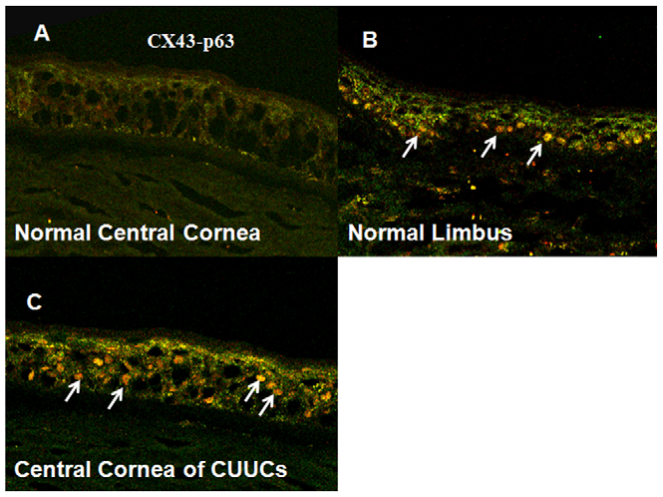
Double immunofluorescent staining of connexin 43 (CX43) and ΔNp63 in a normal central cornea (A), limbus (B) and a CUCC cornea (C) (200×). The epithelial cells of a normal central cornea and the suprabasal epithelial cells of the limbus were positively stained by CX43 antibody (green color); only limbal basal epithelial cells were positively stained by ΔNp63 antibody (orange color and arrows). Both CX43 and ΔNp63 were detected throughout the epithelial cells of CUCC corneas.

Immunohistochemical studies of all CUCC buttons, including 5 eyes from keratoconus patients, 1 eye with corneal ectasia due to previous LASIK surgery and 3 eyes with corneal scars and irregular astigmatism following RK, underwent immunohistochemical staining with mAb 4A4 against ΔNp63, CX 43, and Krt3 monoclonal antibody (Table 2). In the control cornea, the full thickness of the central corneal epithelium was positively stained by the Krt3 and CX43 antibodies but negatively stained by the ΔNp63 antibody (Fig. 1). In contrast, Krt3 and CX43 were positively stained in suprabasal epithelial cells, whereas ΔNp63 only stained the basal epithelial layer of the limbus (Fig. 1). The differences in immunostaining patterns were also demonstrated through double-staining with CX43 and ΔNp63 (Fig. 2). The epithelial cells of the central control cornea and suprabasal epithelial cells of the limbus were positively stained by CX43 antibody; however, only limbal basal epithelial cells were positively stained by ΔNp63 antibody. Both CX43 and ΔNp63 were positively stained in the entire thickness of the epithelial cells of the CUCC (Fig. 2).

**Table 2:** Comparison of immunohistochemical patterns between normal cornea and 8 cases of CUCCs with the markers of corneal epithelial stem/progenitor cells and corneal-type epithelial differentiation.

	Normal limbal basal cells	Normal central corneal epithelial cells	Continuously unstable curvatures of corneas (central cornea)
ΔNp63	Positive	Negative	Positive
Krt3	Negative	Positive	Positive
CX43	Negative	Positive	Positive

**Figure 3:**
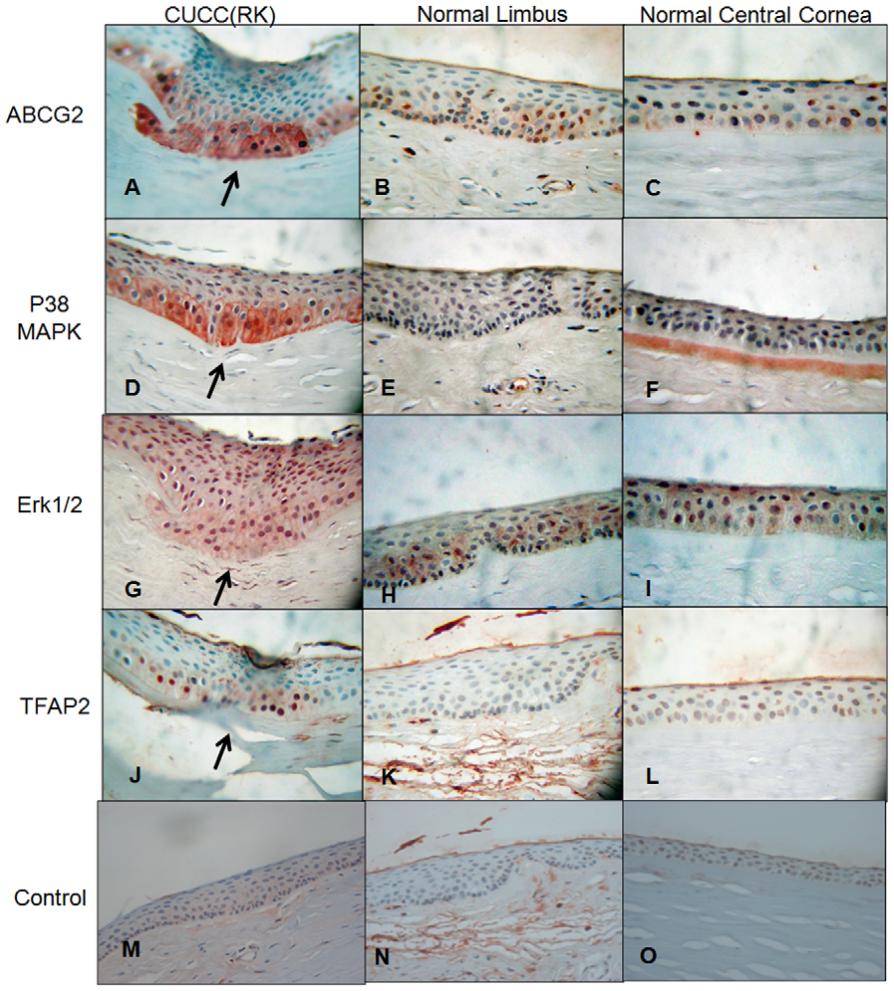
An RK cornea (case 8) immunostained with ABCG2, p38MAPK, Erk1/2, and TFAP2 antibodies (200×). In the limbus of normal cornea, ABCG2 was observed in the basal epithelial cell layers (C). However, the corneal epithelial cells of the central cornea were not stained by ABCG2 antibody (B). In the RK-wounded area of CUCC cornea, ABCG2 staining was obvious in the basal epithelial cell layers (A). Furthermore, p38MAPK (D) and **TFAP2** (J) were also observed in the basal epithelial cells of the wounded area of the RK. The central part of the normal cornea and limbus did not demonstrate expression of P38MAPK (E, F) or **TFAP2** (K, L). **Erk1/2** was not identified in the basal epithelial cells of any of these samples (G, H, I). Moreover, an intact Bowman's membrane was found only in the central part of normal cornea. However, a disrupted Bowman's membrane was found in both the CUCC cornea and the normal limbus. The negative controls are also shown (M, N, O, 100×).

Because of the differences in the immunohistochemical staining patterns observed in our pilot study, we examined the cornea of an RK case with CUCC (case 8) for the expression of ABCG2, p38MAPK, **Erk1/2**, and **TFAP2** (Fig. 3). In the normal limbal cornea, ABCG2 could only be identified in the basal limbal epithelial cells; the central corneal epithelial cells were negative for ABCG2. In the wounded region of the RK cornea, ABCG2, p38MAPK, and **TFAP2** were found in the basal epithelial cells. In contrast, the central normal cornea was negative for ABCG2, P38MAPK, and **TFAP2**. **Erk1/2** was found in the basal epithelial cells of all samples. Moreover, an intact Bowman's membrane was found only in the central normal cornea but not in the RK cornea (arrows in Fig. 3). Disrupted Bowman's membranes and BM were found in all other CUCC corneas.

## Discussion

In the present study, all corneas examined, including those from patients with RK, keratoconus, and corneal ectasia caused by previous LASIK, shared similarities with respect to corneal damage and CUCC symptoms. We found that certain differentiation markers (i.e., Krt3 and CX43) were **not** found in the normal limbal basal epithelium or central corneal epithelium of CUCC patients; rather, these markers were only detected in the central corneal epithelium but not the limbal basal epithelium of a normal cornea. ΔNp63 and ABCG2, the markers of limbal SCs/progenitor cells, were detected in the basal epithelial layers of the limbal epithelium and not in the central corneal epithelium (Tables 2 and 3; Fig. 2 and Fig. 3). In studying case 8, we found that the basal corneal epithelial cells in the RK wound expressed the limbal stem/progenitor markers on disrupted BM and Bowman's membrane, with expression potentially mediated by the p38-MAPK pathway (Fig. 3). A similar finding was also noted in the study by Cheng *et al*., in which the limbal progenitor cell markers could be activated *in vitro* through the same pathway.[41] This phenotypic change in basal epithelial cells might stem from alteration of their niche environment, including the extracellular matrix, or be influenced by the keratocytes beneath the disrupted BM. This concept of an altered niche is compatible with that of SCs in hematopoiesis and other tissues. Notably, *in vitro* systems that support proliferation, differentiation, and survival of distinct progenitor populations have been found to be dependent on factors secreted by stromal cell types and extracellular matrix components. Furthermore, adhesion of basal epithelial cells to the extracellular matrix is mediated by several classes of receptor, the most extensively characterized being integrins. High expression of β1 integrin is required for maintenance of epidermal stem cells, and β1 integrin regulates differentiation of keratinocytes and other cell types through MAP kinase signaling[42] and can directly activate growth factor receptors.[43] Alternatively, the disrupted BM may allow the free diffusion of cytokines, growth factors secreted by the stromal cells to target overlaying epithelial cells, because BM can sequester and modulate the local concentration of factors secreted by stromal cells. Our results are consistent with the notion that maintenance of the niche is essential to maintaining corneal epithelium homeostasis.

**Table 3:** Immunohistochemical patterns between the normal cornea and the RK case with the markers of corneal epithelial stem/progenitor cells and corneal-type epithelial differentiation.

	Normal limbal stem cells	Normal central corneal epithelial cells	Continuously unstable corneal curvatures (central cornea)
Krt3	Negative	Positive	Positive
Cx43	Negative	Positive	Positive
ΔNp63	Positive	Negative	Positive
ABCG2	Positive	Negative	Positive(+++)
p38 MAPK	Negative	Negative	Positive(+++)
AP2	Negative	Negative	Positive
ERK	Positive (+)	Positive (+)	Positive (+++)

In an RK wound, the altered anatomical structure of the BM is very similar to that of the limbus, which serves as a niche for limbal SCs.[44] The α1–αl2 (IV) chains of type IV collagen in the BM around epithelial plugs are present only in the limbal BM, and α3 and α4 chains are very rare or absent in these areas, in comparison with non-scarred areas. Krt 3 also presents a limbal-like, suprabasal expression pattern in the plug epithelium. The stroma around the scars accumulate tenascin-C, fibrillin-1, types VIII and XIV collagen, all of which are absent in normal corneal BM and extracellular matrices. This particular alteration of the BM possibly provides **an altered niche** that enables the central corneal basal epithelium to undergo de-differentiation and assume a phenotype resembling that **of** corneal stem/progenitor cells.

In addition, epithelial-mesenchymal interactions mediated through humoral factors, such as cytokines, chemokines, and growth factors secreted by epithelial cells and/or the underlying keratocytes, have important roles in the maintenance of corneal integrity and function as well as in wound healing.[45]–[47] The cellular factors involved in such crosstalk include transforming growth factor-α (TGF-α), TGF-β, interleukin-1 (IL-1)[48], [49], platelet-derived growth factor (PDGF)[50], keratinocyte growth factor (KGF)[51], hepatocyte growth factor (HGF)[52], and others. Cheng *et al*. showed that KGF and HGF are two paracrine factors that regulate the proliferation, migration and differentiation of the limbal epithelial cells, and they found that KGF is a more potent growth stimulator of epithelial outgrowth than HGF. Epithelial outgrowth from cells treated with HGF or KGF showed similar expression patterns for Krt3 and Krt14.[41] However, p63 was highly expressed by KGF-treated limbal epithelial sheets but not by those treated with HGF. Kinase inhibitor studies showed that induction of ΔNp63αexpression by KGF is mediated via the p38 pathway.[41] Their results and ours indicate that phosphorylation and activation of the Ras/Raf/mitogen-activated protein kinase (MAPK) pathway leads to phosphorylation of regulatory proteins and transcription factors, culminating in cell migration, proliferation, and/or differentiation both in basal epithelial cells of the RK wound and cells grown from limbal explant culture.

In conclusion, we propose that the phenotypic changes in basal corneal epithelial cells in the CUCC, which expresses the limbal SC markers on disrupted BM and Bowman's membrane (potentially through the p38-MAPK pathway), might be due to an alteration of their niche, including the extracellular matrix and the BM.
